# Development and Carcinogenesis: Roles of GATA Factors in the Sympathoadrenal and Urogenital Systems

**DOI:** 10.3390/biomedicines9030299

**Published:** 2021-03-15

**Authors:** Takashi Moriguchi

**Affiliations:** Division of Medical Biochemistry, Faculty of Medicine, Tohoku Medical and Pharmaceutical University, Sendai 983-8536, Japan; moriguchi@tohoku-mpu.ac.jp

**Keywords:** GATA2, GATA3, sympathoadrenal, urogenital, neuroblastoma, urothelial cancer, gynecologic cancer

## Abstract

The GATA family of transcription factors consists of six proteins (GATA1-6) that control a variety of physiological and pathological processes. In particular, GATA2 and GATA3 are coexpressed in a number of tissues, including in the urogenital and sympathoadrenal systems, in which both factors participate in the developmental process and tissue maintenance. Furthermore, accumulating studies have demonstrated that GATA2 and GATA3 are involved in distinct types of inherited diseases as well as carcinogenesis in diverse tissues. This review summarizes our current knowledge of how GATA2 and GATA3 participate in the transcriptional regulatory circuitry during the development of the sympathoadrenal and urogenital systems, and how their dysregulation results in the carcinogenesis of neuroblastoma, renal urothelial, and gynecologic cancers.

## 1. Introduction

The mechanistic aspects underlying cell lineage-specific differentiation have been extensively studied over the last several decades. Accumulating studies have elucidated how particular regulatory network leads to the activation of a specific differentiation program that determines the fate of each cell lineage. One of the key representative factors orchestrating such regulatory programs is the GATA family of transcription factors, which comprises six zinc-finger proteins (GATA1 through GATA6) and regulates various cell differentiation and proliferation processes [1,2]. GATA2 is known as a key regulator of hematopoietic development and the maintenance of hematopoietic stem cells [3]. Recent genetic and clinical studies have established that haploid deficiency of GATA2 predisposes patients to hematopoietic malignancy and primary immune deficiency syndrome [4,5]. GATA3 was originally identified as a master regulator of T-lymphocyte-lineage development [6,7,8]. Later, it was found that GATA3 performs fundamental functions in the development of the sympathetic ganglia, adrenal medulla, kidney, inner ear, mammary gland, and parathyroid gland [9,10,11,12,13,14,15,16]. Haploid GATA3 deficiency leads to a clinical triad represented by Hypoparathyroidism, Deafness, and Renal anomaly (HDR syndrome), underscoring the essential function of GATA3 in development of these tissues [17,18]. Decades of research have demonstrated that GATA2 and GATA3 are coexpressed in a number of tissues, including the urogenital and sympathoadrenal systems, in which both factors function distinctly or redundantly in developmental processes and in the function of mature tissues. Furthermore, GATA2 and GATA3 have also been associated with carcinogenesis in a series of organs in which each factor plays a role in dysregulated cellular proliferation. In this review, we summarize recent insights regarding the physiological functions of GATA2 and GATA3 in the developing urogenital and sympathoadrenal systems, and how alteration of their activity or expression pattern promotes carcinogenesis.

## 2. Review Design

We screened PubMed, EMBASE, and Cochrane Library databases for relevant literature from Apr 1995 to Jan 2021 and subjected the resulting references to the review. The search was limited to articles written in English and used the following search keywords: “GATA2”, “GATA3”, “urogenital”, “sympathoadrenal”, “neuroblastoma”, “gynecological cancer”, and “urothelial cancer”. This review includes studies elucidating physiological functions of GATA2 and GATA3 in the developing urogenital and sympathoadrenal systems using reliable in vivo and in vitro experimental systems. Authentic studies demonstrating clinical relevance of GATA2 and GATA3 dysregulation in neuroblastomas and urogenital cancers were also involved.

## 3. GATA2 and GATA3 in Sympathetic and Adrenal Chromaffin Cell Development

**Figure 1:**
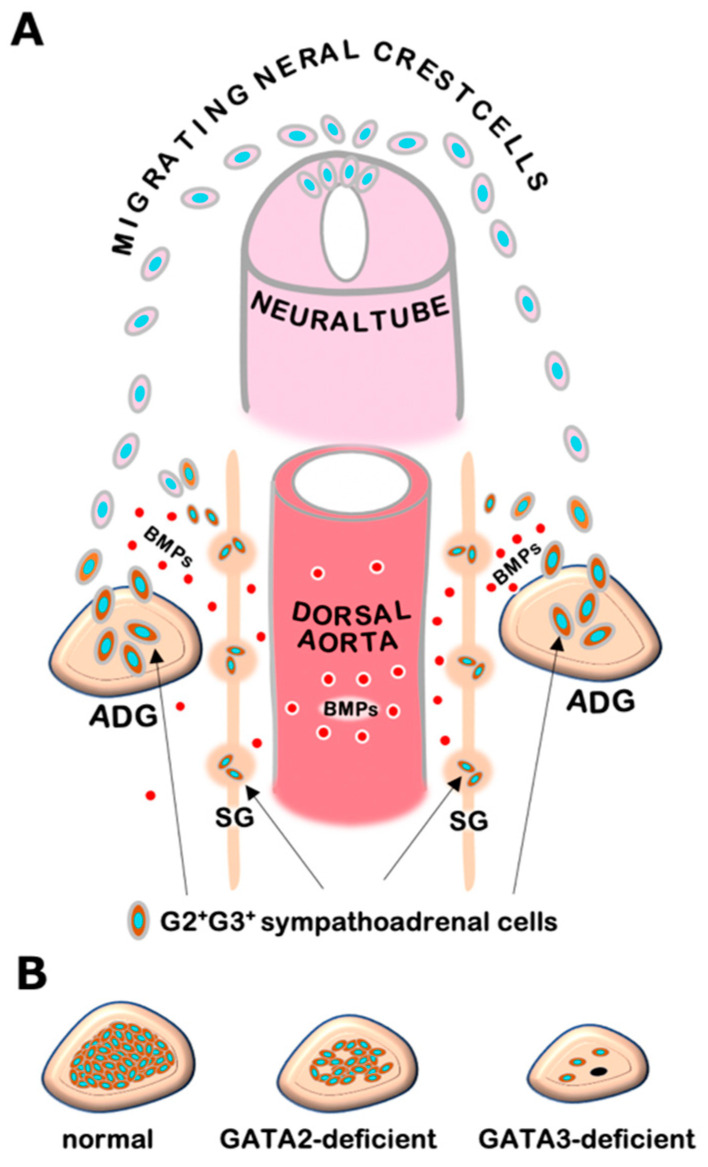
(**A**) Neural crest cells migrating from the dorsal neural tube undergo differentiation to sympathetic progenitor cells in response to bone morphogenetic proteins (BMPs) secreted from embryonic dorsal aorta. The sympathetic progenitors, which express GATA2 and GATA3, migrate to sympathetic ganglia (SG) and adrenal gland (ADG), and thereafter give rise to sympathetic neuronal cells and adrenal medullary chromaffin cells. (**B**) GATA3-deficient embryos show a significant decrease in adrenal chromaffin cells (right), while sympathoadrenal cell-specific GATA2-deficient embryos exhibit a modest decrease in chromaffin cells (middle) in comparison with normal adrenal gland in wild-type mice (left).

The sympathoadrenal system contains sympathetic ganglion neurons and adrenal chromaffin cells, both of which are derived from a common progenitor from the neural crest. Genetic studies in mice and other model organisms have revealed that a series of tissue-specific transcription factors participate in the regulatory network of sympathoadrenal development [19]. In early embryogenesis, neural crest cells that arise from the dorsal neural tube undergo differentiation into sympathetic progenitor cells and express key regulators including Phox2a, Phox2b, HAND2, GATA2, and GATA3 in response to bone morphogenetic proteins (BMPs) secreted from the embryonic dorsal aorta [9,11,12,20] (Figure 1A). These sympathetic progenitors acquire catecholaminergic characteristics typified by the expression of catecholamine biosynthesis enzymes, i.e., tyrosine hydroxylase (TH) and dopamine b-hydroxylase (DBH). Subsequently, the sympathetic progenitors migrate to their final destinations and give rise to definitive sympathetic neurons and adrenal chromaffin cells [12]. GATA3 plays a fundamental role in the developmental program of sympathoadrenal lineage cells and directs the expression of noradrenergic marker genes [9,11]. Thus, GATA3 deficiency leads to a significant loss of TH and DBH expression as well as developmental defects both in sympathetic neurons and adrenal chromaffin cells [9,11,20]. Catecholamines produced in the sympathoadrenal system are essential for cardiovascular development in mouse embryos. TH- or DBH-deficient mice show systemic catecholamine deficiencies that result in embryonic lethality due to cardiovascular failure, which is pharmacologically rescued by administering catecholamine analogs to pregnant dams in drinking water [21,22,23]. The GATA3 null-deficient mice exhibits similar heart failure and dies by the mid-gestational stage (e10.5 in mouse embryos). As in the TH- and DBH-deficient mice, the embryonic lethality of GATA3-deficient mice can be prevented pharmacologically by administering adrenaline analogs [9]. Additionally, transgenic GATA3 complementation in sympathoadrenal lineage cells is capable of decreasing lethality and restoring sympathoadrenal development in the GATA3-deficient mice [11]. These results underscore the essential requirement of GATA3 for adrenaline biosynthesis and sympathoadrenal development [9,11].

It has been shown that GATA2 could be important for the early sympathetic neuronal development in chick embryos [20]. Forcible expression of GATA2 induces adrenergic neurogenesis in the chick neural tube. In mouse embryos, GATA2 expression is detected in developing sympathetic progenitors [20]. However, the expression pattern of GATA2 and the roles played by GATA2 in adrenal chromaffin cells have never been elucidated. Recently, we demonstrated that GATA2 is highly specifically expressed in the adrenal chromaffin cells of adult mice [24]. Sympathoadrenal cell lineage-specific deletion of GATA2 exploiting neural crest-specific Wnt1-Cre mice (GATA2 NC-CKO) led to decreased TH-expressing adrenal chromaffin cells and resulted in mid-gestational lethality [24]. The lethality of the GATA2 NC-CKO embryos was associated with the diminished catecholaminergic trait and was also partially rescued by administering adrenaline analogs to the pregnant dam [24]. These results indicate that like GATA3, GATA2 also participates in adrenaline biosynthesis processes in adrenal chromaffin cells.

GATA2 NC-CKO embryos show relatively late embryonic lethality (e14.5) with a modest decrease in chromaffin cells, while GATA3-deficient mice undergo early embryonic death (e10.5) with significant decreases in sympathetic neurons and adrenal chromaffin cells [9,24] (Figure 1B). Furthermore, in contrast to GATA3-deficient mice, GATA2 NC-CKO mice do not show apparent cardiovascular defects [9,24]. These results suggest that GATA3 plays a more important role than GATA2 in developing sympathoadrenal cells. It was reported that GATA2 mRNA expression was decreased in GATA3-deficient sympathetic progenitor cells [11,20]. In contrast, GATA3 expression was not significantly affected in GATA2-deficient chromaffin cells [24]. These results indicate that GATA2 may function hierarchically downstream of GATA3. Further analysis of the interregulatory relationships between GATA2 and GATA3 and their distinct and cooperative functions in the sympathoadrenal system would extend our understanding of molecular basis underlying sympathoadrenal development.

## 4. Neuroblastomas and GATA Factors

Neuroblastoma is one of the most common solid tumors in children and arises due to disordered development of the sympathetic nervous system from neural crest cells [25]. Activation of oncogenes, including MYCN, ALK, and TERT, has been deemed a major driver of neuroblastoma oncogenesis [25]. While expression of GATA2 and GATA3 in human neuroblastoma with relatively low malignant potential has been previously reported, their functional roles in neuroblastomas have not been assessed [26].

**Figure 2:**
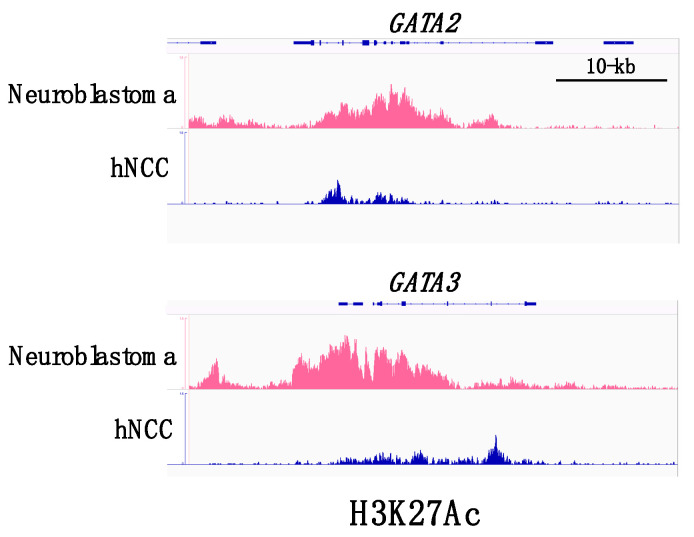
ChIP-seq (chromatin immune-precipitation sequencing) profiles for H3K27ac binding at *GATA2* and *GATA3* super-enhancers. *GATA2* and *GATA3* loci in neuroblastoma cells are associated with dense accumulation of H3K27ac in comparison with the loci in normal human neural crest cells (hNCC). Datasets are derived from SRX2911572 (human neural crest cells) and SRX5705566 (human neuroblastoma cells).

A recent comprehensive analysis delineated the transcriptional regulatory network in a panel of neuroblastoma cell lines and demonstrated that the majority of the neuroblastoma cell lines (18/25) exhibit a noradrenergic identity characterized by enhanced regulatory circuits involving PHOX2A, PHOX2B, HAND2, GATA2, and GATA3 [27]. The gene loci encoding these transcription factors tended to be associated with super-enhancers (Figure 2). Super-enhancers consist of large clusters of enhancers with dense accumulation of enhancer chromatin signatures, including monomethylation of histone H3 at lysine 4 (H3K4me1) and acetylation of histone H3 on lysine 27 (H3K27ac), as well as binding of lineage-specific master transcription factors. Cancer cells often acquire super-enhancers at oncogene loci and induce aberrant expression of the oncogenic factor that determines the cancer cell identity [28]. Since GATA2 and GATA3 are both involved in a complex transcription factor network regulating sympathoadrenergic traits [9,11,24], the frequent occurrence of super-enhancers in GATA2 and GATA3 loci appears to underlie the sympathetic noradrenergic cell identity of neuroblastoma cells. Furthermore, it is conceivable that GATA2 and GATA3 play a fundamental role in the genesis of neuroblastoma, given their roles in normal sympathoadrenal development. Consistent with this hypothesis, GATA3 was reported to be a first-line immunohistochemical marker to facilitate the pathological diagnosis of neuroblastic tumors [29].

*Gata3 cis*-regulatory studies using transgenic reporter mouse assays have revealed that even a 662-kb mouse *Gata3* YAC containing 451-kb and 211-kb of 5′ and 3′ flanking sequences, respectively, was missing the regulatory element(s) that confer expression in sympathoadrenal system organs [30]. Neuroblastoma-associated super-enhancers are distributed among a broad range of genomic loci of human *GATA2* and *GATA3* [27]. Therefore, it would be of particular interest to address whether the constituents of the oncogenic super-enhancers in the *GATA3* locus could function as sympathoadrenal tissue-specific *cis*-regulatory elements in the physiological context. By identifying the *cis*-regulatory elements governing the sympathoadrenal tissue specificity of GATA factors and elucidating their detailed activities, we could understand the complex regulatory network that underlies both normal sympathoadrenal development and pathogenic neuroblastomagenesis.

## 5. GATA3 and DNA Methylation in Neuroblastoma

Recent genome-wide studies have characterized broad alterations in DNA methylation status in a series of neuroblastoma cell lines [31]. In these neuroblastoma cell lines, the DNA methylation level of the *GATA3* gene locus was lower than that in normal human neural crest cells [32]. Subsequent analysis of clinical relevance revealed that high *GATA3* expression tended to be associated with poor prognosis, indicating a close correlation between *GATA3* expression levels and the clinical properties of neuroblastomas [32]. As anticipated, *GATA3* siRNA (small interfering RNA) knockdown in neuroblastoma cell lines inhibited proliferation and increased apoptosis [31]. These results demonstrate that GATA3 functions in the transcriptional network that promotes neuroblastoma proliferation, which is subjected to epigenetic regulation via DNA methylation. In this regard, the GATA3-specific deoxyribozyme (single-stranded synthetic DNA antisense molecules) that has been successfully applied to allergic asthma patients in recent phase 2a clinical trial may exert some efficacy against neuroblastoma [33]. Considering that GATA2 also participates in sympathoadrenal development, it would be important to elucidate whether GATA2 plays a role in the genesis and maintenance of neuroblastoma, as does GATA3, under similar regulatory mechanisms.

Identification of direct target genes regulated by GATA2 and/or GATA3 for neuroblastoma proliferation has become increasingly important. In a series of neuroblastoma cell lines, GATA3 was found to positively regulate cyclin D1 transcription by directly binding its promoter sequences [34]. Cyclin D1 maintains cellular proliferation in the vast majority of neuroblastoma tumors, suggesting that GATA3 exerts oncogenic potential by transactivating cyclin D1 in neuroblastomas. Collectively, these results suggest that GATA3 plays a fundamental role in neuroblastomagenesis, and thus the transcriptional network involving GATA3 can be a hopeful target for novel neuroblastoma therapies.

## 6. GATA2 and GATA3 in Urogenital Development

**Figure 3:**
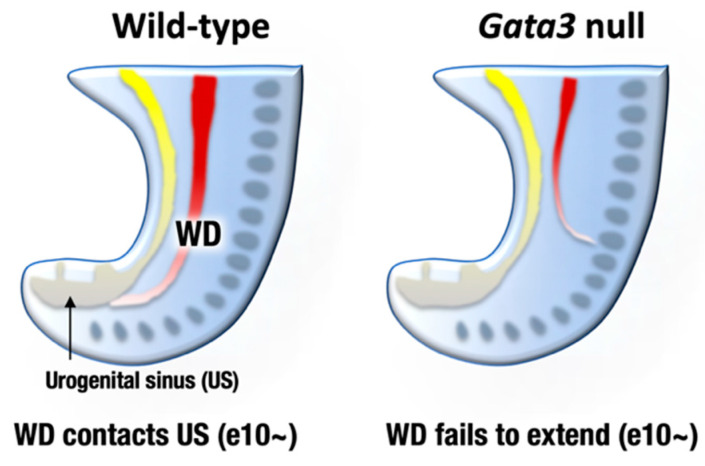
By embryonic day 9–10, mesonephric ducts (WD, Wolffian ducts) extend posteriorly to reach the urogenital sinus (US) that later give rise to bladder and urethra (**left**). *Gata3*-deficient mouse embryos fail to extend nephric duct to the US. The atretic remnant of the nephric duct sometimes extends to aberrant direction (**right**). Consequently, metanephric kidney development is disturbed in the *Gata3*-deficient embryos.

**Figure 4:**
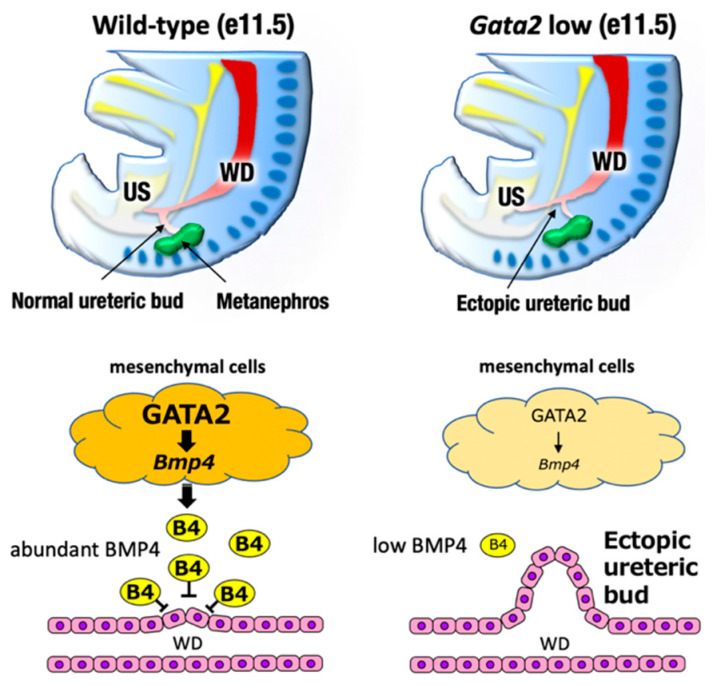
The definitive kidney development begins around e11 when the ureteric bud (UB) sprouts from the posterior WD. Bone morphogenetic protein 4 (BMP4), which is secreted by the urogenital mesenchymal cells surrounding the UB, prevents the aberrant outgrowth of the UB from rostral ectopic sites on the WDs. GATA2-low mice failed to express sufficient level of BMP4. Consequently, the ureteric budding is mislocated in the rostral position of Wolffian duct, which compromise the ureter-bladder connection.

The urogenital system comprises the kidney, reproductive system, and urinary and genital tracts, all of which are derived from embryonic intermediate mesoderm [35]. Urogenital development starts with the bilateral formation of the Wolffian ducts (WDs, also known as the mesonephric ducts) in the intermediate mesoderm (Figure 3, left). The WDs extend posteriorly to contact the urogenital sinus, which later gives rise to the bladder and urethra. Development of the definitive kidney begins approximately e11 when a ureteric bud (UB) sprouts from the posterior WD in response to glial cell-derived neurotrophic factor (GDNF), which is secreted from the adjacent mesenchyme [35] (Figure 4, left). Subsequently, the UB undergoes extensive bifurcation and branching in the developing metanephros and gives rise to the collecting ducts and the distal renal tubules of the definitive kidney. Bone morphogenetic protein 4 (BMP4), which is secreted by the urogenital mesenchymal cells surrounding the UB, prevents the aberrant outgrowth of the UB from rostral ectopic sites on WDs by suppressing GDNF signaling (Figure 4, left). As for reproductive systems, WDs give rise to the upper male genital tract, such as the epididymis, vas deferens, and seminal vesicles. In females, Müllerian duct gives rise to the oviduct and uterus [36].

GATA2 and GATA3 are both expressed in the developing mouse urogenital system, while their expression patterns are different. GATA2 is expressed both in the mesenchymal and epithelial tissues of the WDs and UB, whereas GATA3 is specifically expressed in the epithelial cells of the urogenital anlagen [37,38,39]. GATA3 promotes WD extension and insertion into the urogenital sinus (primordium of the bladder and urethra) at e10 and afterwards [38,40,41] (Figure 3, left). Subsequently, GATA3 induces c-Ret expression in the UB and facilitates the GDNF-mediated UB outgrowth and branching morphogenesis of the definitive kidney [42]. Indeed, pharmacologically rescued *Gata3*-deficient embryos exhibited fatal defects in nephric duct formation. The atretic remnant of the nephric duct sometimes extended in an abnormal direction and terminated within the lateral body wall [38] (Figure 3, right). Consequently, *Gata3*-deficient mice failed to form the definitive metanephric kidney [9,38]. Additionally, *Gata3*-deficient female and male late embryos lacked genital organs, i.e., uteri or epididymides/vas deferens [38].

In humans, loss of the haploid *GATA3* allele predisposes patients to an inherited disease manifesting as renal anomaly that ranges widely from vesicoureteral reflux and renal hypoplasia to complete agenesis (i.e., HDR syndrome) [18]. While *Gata3* heterozygous adult mice did not show major genitourinary defects, early renal patterning defects, such as additional ureteric buds, were infrequently found in *Gata3* heterozygous embryos [38]. These minor mispatternings in *Gata3* heterozygous embryos suggest that human GATA3 haploinsufficiency is partially reproduced in the mouse model.

## 7. GATA2 and Hydronephrosis

The participation of GATA2 in urinary tract development was first demonstrated when the lethal hematopoietic deficiency in *Gata2*-deficient embryos was rescued with transgenic GATA2 complementation in hematopoietic lineage cells. Transgenic GATA2 expression directed by the 271-kbp *Gata2* yeast artificial chromosome (YAC) restored normal hematopoietic development in *Gata2*^−/−^ embryos and successfully rescued embryonic lethality up to the perinatal stage [43]. However, the YAC-rescued compound *Gata2* mutant neonates failed to develop a proper ureter–bladder connection and consequently succumbed to urinary tract anomalies such as hydronephrosis and megaureter [43].

Exploiting cellular and mouse genetic analyses, we found that GATA2 positively regulated BMP4 expression in the caudal urogenital mesenchyme [39]. A proper level of BMP4 expression was a prerequisite for the antero-caudally correct sprouting of the UB and the subsequent correct ureter–bladder connection [39]. GATA2 hypomorphic mutant mice in which GATA2 expression was diminished to less than the haploid level failed to express sufficient levels of BMP4 in the urogenital mesenchyme [44]. Consequently, ureteric budding was mislocated in the rostral position of the Wolffian duct, which compromised the ureter–bladder connection and thereby led to megaureter resembling human congenital anomalies of the kidney and the urinary tract (CAKUT), which is one of the most frequent human birth defects [39,44] (Figure 4, right). It was reported that there were multiple distal enhancers controlling urogenital system-specific GATA2 expression at 75–113 kbp 3′ to the *GATA2* structural gene [37]. Transgenic GATA2 or BMP4 expression driven by the *Gata2* distal urogenital enhancer significantly restored the ectopic outgrowth of the UB and therefore remedied the urinary tract anomaly observed in *Gata2* hypomorphic mutant mice [39]. Because urogenital *Gata2* enhancers contain multiple evolutionarily conserved GATA binding sites, GATA2 likely autoregulates itself in the urogenital mesenchyme, as is the case in hematopoietic lineage cells [1,39]. Recently, it was reported that ureteric mesenchyme-specific conditional GATA2 deletion mice also phenocopied similar hydroureter anomalies [45]. Collectively, these results demonstrate that GATA2 plays a crucial role in regulating ureter positioning in the renal mesenchyme by acting through BMP4 signaling.

## 8. GATA3 and Urogenital Cancers

Urothelial cancer, which is also known as transitional cell carcinoma (TCC), develops from transitional epithelial cells lining the urethra, bladder, ureters, and renal pelvis. In particular, the incidence and mortality of bladder cancer have rapidly increased in the past few decades. It was reported that GATA3 was expressed in most urothelial carcinomas, consistent with its physiological expression profiles and biological roles in the urinary tract [46,47]. Since then, GATA3 has been deemed a useful marker for detecting primary and metastatic urothelial carcinomas. Closer examination, however, demonstrated that GATA3 expression was more frequently detected in low-grade urothelial carcinomas than in high-grade cases [48]. A simple experiment demonstrated that forced expression of GATA3 diminished the proliferation of a bladder cancer cell line, suggesting that GATA3 has tumor suppressor activity in the bladder cancer cells [47]. These observations imply that GATA3 might play a similar role as in breast cancer. In the normal mammary gland, GATA3 is required for luminal epithelial cell differentiation, and its expression is progressively lost during luminal breast cancer progression as cancer cells acquire an immature phenotype. Importantly, forced GATA3 expression in GATA3-negative, immature breast carcinoma cells induced tumor differentiation and reduced malignant properties [49,50]. These findings demonstrate the exquisite ability of GATA3 to attenuate the malignant properties of bladder cancer as well as breast cancer and raise the possibility that GATA3 or its downstream genes could be therapeutic targets in cancer therapy.

In renal cell carcinomas, GATA3 is frequently expressed in chromophobe renal cell carcinomas, renal oncocytoma, and clear cell papillary renal cell carcinoma [51,52]. These types of tumors are often derived from the collecting ducts that is the physiological GATA3 expression sites in nephrons, implying GATA3 partake in the cell-intrinsic carcinogenic mechanism [13,53,54].

As for female reproductive systems, GATA3 can be a useful immunohistochemical marker for diagnosis of mesonephric-like carcinoma of the endometrium in the uterus and endometriosis [55,56,57]. Since GATA3 is developmentally required for genesis of Müllerian duct-derived organs such as the uterus, it is important to address whether alteration of GATA3 activity participates in uterine carcinogenesis.

## 9. Conclusions

The roles played by GATA2 and GATA3 in sympathoadrenal and urogenital differentiation add to the growing body of evidence implicating the GATA family of transcription factors as key regulators of cell fate specification and maintenance. In carcinogenesis, activity of GATA factors can be altered by a multitude of mechanisms that impair function or reduce expression. These alterations might direct GATA factors either toward tumor promotion or tumor suppression, depending on the context. While GATA3 serves as a diagnostic marker for urothelial cancers, forced expression of GATA3 diminishes malignant propensity of bladder cancer cells. In contrast, the results thus far have shown that GATA3 solely functions as an oncogenic factor by transactivating cyclin D1 expression in neuroblastomas. A better understanding of how GATA2 and GATA3 function as oncogenes or tumor suppressors is of prime importance for the treatment of neuroblastoma and urothelial cancers and will shed further light on their roles as prognostic factors. Importantly, similar to other transcription factors, GATA factors function in combination with other coregulators including acetyl transferases, deacetylases, and methyltransferases to confer specific patterns of gene regulation. Manipulating the activity of these cofactors by applying pharmacological compounds is a challenging and rewarding therapeutic avenue.

## Data Availability

ChIP-seq data sets are derived from SRX2911572 (human neural crest cells) and SRX5705566 (human neuroblastoma cells).
